# Usability of Wearable Devices to Remotely Monitor Sleep Patterns Among Patients With Ischemic Heart Disease: Observational Study

**DOI:** 10.2196/14508

**Published:** 2020-04-07

**Authors:** Michael Fortunato, Srinath Adusumalli, Neel Chokshi, Joseph Harrison, Charles Rareshide, Mitesh Patel

**Affiliations:** 1 Crescenz Veterans Affairs Medical Center Philadelphia, PA United States; 2 University of Pennsylvania Philadelphia, PA United States

**Keywords:** sleep, wearable devices, ischemic heart disease

## Abstract

**Background:**

There is growing interest in using wearable devices to remotely monitor patient behaviors. However, there has been little evaluation of how often these technologies are used to monitor sleep patterns over longer term periods, particularly among more high-risk patients.

**Objective:**

The goal of the research was to evaluate the proportion of time that patients with ischemic heart disease used wearable devices to monitor their sleep and identify differences in characteristics of patients with higher versus lower use.

**Methods:**

We evaluated wearable device data from a previously conducted clinical trial testing the use of wearable devices with personalized goal-setting and financial incentives. Patients with ischemic heart disease established a sleep baseline and were then followed for 24 weeks. The proportion of days that sleep data was collected was compared over the 24 weeks and by study arm. Characteristics of patients were compared to groups with high, low, or no sleep data.

**Results:**

The sample comprised 99 patients with ischemic heart disease, among which 79% (78/99) used the wearable device to track their sleep. During the 6-month trial, sleep data were collected on 60% (10,024/16,632) of patient-days. These rates declined over time from 77% (4292/5544) in months 1 and 2 to 58% (3188/5544) in months 3 and 4 to 46% (2544/5544) in months 5 and 6. Sleep data were collected at higher rates among the intervention group compared with control (67% vs 55%, *P*<.001). In the main intervention period (months 3 and 4), patients with higher rates of sleep data were on average older (*P*=.03), had a history of smoking (*P*=.007), and had higher rates of commercial health insurance (*P*=.03).

**Conclusions:**

Among patients with ischemic heart disease in a physical activity trial, a high proportion used wearable devices to track their sleep; however, rates declined over time. Future research should consider larger evaluations coupled with behavioral interventions.

**Trial Registration:**

ClinicalTrials.gov NCT02531022; https://clinicaltrials.gov/ct2/show/NCT02531022

## Introduction

Shorter sleep duration and poor sleep quality have been demonstrated to be associated with higher rates of all-cause mortality, cardiovascular disease, hypertension, and obesity [1,2]. Most of these evaluations have relied on patient self-report of sleep patterns, which requires effort from the patient and can be subject to reporter bias. There has been growing interest in using wearable devices to passively collect data on patient behaviors [3,4]. However, there has been little evaluation of how often these technologies are used to remotely monitor sleep patterns, particularly among more high-risk patients and over longer term periods.

There are more than 50 different wearable devices that promote the ability to track sleep patterns [5]. In a recent review article, 43 articles identified studied how these wearables were used to track sleep patterns [6]. More than half were focused on validating sleep accuracy; few were focused on monitoring sleep during behavioral interventions. Several studies have evaluated the use of these devices over 24-hour periods [7,8]. Others have evaluated tracking sleep during behavioral interventions focused on physical activity [6,9], but these have typically been for periods of 3 months or less.

In this study, we used data from a behavioral intervention focused on increasing physical activity among ischemic heart disease patients over 24 weeks. The objective was to evaluate the proportion of time that patients with ischemic heart disease used wearable devices to monitor their sleep and identify differences in characteristics of patients with higher versus lower use.

## Methods

The sample comprised patients with ischemic heart disease who used wearable devices to establish baseline sleep levels during the ACTIVE REWARD (A Clinical Trial Investigating Effects of a Randomized Evaluation of Wearable Activity Trackers with Financial Rewards) trial, a previously conducted 24-week randomized clinical trial focused on increasing physical activity [10]. All patients established baseline daily step counts and were then randomly assigned to passive monitoring or an intervention that used personalized goal-setting and financial incentives to increase physical activity levels. In this study, we included patients who established baseline levels of sleep during the run-in period (99/105 patients). Financial incentives for meeting step goals were offered to the intervention group for 16 weeks followed by 8 weeks without incentives. Patients were asked to use the wearable devices (Shine, Misfit) during the day and night. The wearable did not require charging (with a battery life longer than 6 months), was waterproof, and displayed progress toward the step goal (rather than the actual number of steps) on its display. Patients could use the mobile app to obtain the actual number of steps. The Shine has been found to be reliable for monitoring sleep duration when compared with polysomnography [11].

To evaluate use of wearable devices for tracking sleep data, we analyzed the proportion of patient-days data that were collected overall and during 8 week increments throughout the three trial phases. Intervention patients had 3 phases: ramp-up phase with incentives (valued at $2 per day) and gradually increasing step goals, maintenance phase with incentives and static step goals, and a follow-up phase with static step goals but no incentives. We compared patient characteristics for groups of patients with different levels of overall data collection above and below half of the study period duration (more than 50% of days with data, less than 50% of days with data, no data).

This study was approved by the University of Pennsylvania institutional review board, and patients provided informed consent. The study was registered with ClinicalTrials.gov [NCT02531022]. Analyses were conducted in SAS version 9.4 (SAS Institute Inc).

## Results

The sample comprised 99 patients with ischemic heart disease, with 79% (78/99) using the wearable device to track their sleep. During the 6-month trial, sleep data were collected on 60% of patient-days (Table 1). These rates declined over time from 77% (10,024/16,632) in months 1 and 2 to 58% (3188/5544) in months 3 and 4 to 46% (2544/5544) in months 5 and 6. Sleep data were collected at higher rates among the intervention group compared with control (67% vs 55%; *P*<.001). In the main intervention period (months 3 and 4), patients with higher rates of sleep data were on average older, were less likely to be actively smoking, and had higher rates of private health insurance (Table 2).

**Table 1:** Proportion of patient-days that sleep data was collected by period and arm.

Trial phase	Control (n=2912), n (%)	Intervention (n=2632), n (%)
Ramp-up period: weeks 1-8	2170 (75.52)	2122 (80.62)
Maintenance period: weeks 9-16	1444 (49.59)	1744 (66.26)
Follow-up period: weeks 17-24	1153 (39.59)	1391 (52.85)

**Table 2:** Patient characteristics by use of wearable devices to track sleep. Sleep data are based on the main intervention period (weeks 9 to 16) of the trial.

Characteristics			≥50% sleep data collected (n=60)	<50% sleep data collected (n=18)	No sleep data collected (n=21)	*P* value
**Sociodemographics**						
	Age in years, mean (SD)		62 (9.2)	55.1 (12.4)	55.8 (11.5)	.03
	Male, n (%)		40 (67)	13 (72)	15 (71)	.86
	**Race/ethnicity, n (%)**					.32
		White non-Hispanic	49 (82)	11 (61)	14 (67)	
		Black non-Hispanic	8 (13)	5 (28)	6 (29)	
		Other	3 (5)	2 (11)	1 (5)	
	**Education, n (%)**					.77
		Some high school	3 (5)	2 (11)	1 (5)	
		High school graduate	12 (20)	5 (28)	4 (19)	
		Some college or specialized training	13 (22)	3 (17)	8 (38)	
		College graduate	31 (52)	8 (44)	8 (38)	
		Missing	1 (2)	0 (0)	0 (0)	
	**Marital status, n (%)**					.37
		Single	12 (20)	5 (28)	6 (29)	
		Married	40 (67)	8 (44)	13 (62)	
		Other	8 (13)	5 (28)	2 (10)	
	**Insurance, n (%)**					.03
		Private	35 (58)	5 (28)	9 (43)	
		Medicare	23 (38)	9 (50)	11 (52)	
		Medicaid	1 (2)	4 (22)	1 (5)	
		Military	1 (2)	0 (0)	0 (0)	
	**Annual household income, n (%)**					.74
		Less than $50,000	20 (33)	9 (50)	7 (33)	
		$50,000 to $100,000	12 (20)	4 (22)	6 (29)	
		Greater than $100,000	18 (30)	4 (22)	6 (29)	
		Missing	10 (17)	1 (6)	2 (10)	
**Baseline measures**						
	Baseline step count, mean (SD)		7214.5 (3618.2)	6617.7 (2584.1)	5481.2 (1808.4)	.13
	Body mass index, mean (SD)		30.1 (5.9)	29.6 (5.7)	32 (6.2)	.35
	Diabetes, n (%)		16 (27)	4 (22)	11 (52)	.06
	Hypertension, n (%)		49 (82)	16 (89)	17 (81)	.75
	Hyperlipidemia, n (%)		51 (85)	14 (78)	16 (76)	.59
	**Smoking history, n (%)**					.007
		Nonsmoker	30 (50)	10 (56)	5 (24)	
		History of smoking	29 (48)	5 (28)	11 (52)	
		Actively smoking	1 (2)	3 (17)	5 (24)	

## Discussion

### Principal Findings

There is growing evidence that our sleep patterns influence our longer term health, with poor sleep associated with higher risk for cardiovascular disease [1,2]. Therefore, new ways to collect data on an individual’s sleep could be important to help inform the design of future interventions. To our knowledge, this is one of the first studies to evaluate sleep data collected by wearable devices in a longer 24-week clinical trial.

Our findings reveal several important insights. First, a high proportion of patients used wearable devices to track their sleep; however, rates declined over time. Yet similar to a previous study [9], it is important to recognize that patients were enrolled in a trial focusing on physical activity, not sleep. Therefore, while rates of sleep data were high, they could be increased with a greater intervention emphasis on sleep. Second, we found that sleep data were collected at higher rates among patients in the intervention arm relative to control. This may be because engagement was more related to behavioral reasons (ie, motivation vs loss of interest over time) rather than technical issues (eg, device failure). Taken together, this indicates that use of wearable devices may increase by combined provision of the technology with a behavior change strategy. A recent review suggested that more research is needed on using wearables to monitor sleep in behavioral interventions [6]. Third, use varied across several patient characteristics. Notably, use was significantly higher among older patients, those with a history of smoking, and those with commercial health insurance. This indicates that sleep data may be obtainable from high-risk, older patient groups. Similar results were seen in a shorter, 24-hour study [7], and our findings demonstrate similar insights over a 24-week period. These may need to be investigated in future trials with larger and more diverse populations.

### Strengths and Limitations

This study had several strengths. First, most recent studies have focused on comparing the accuracy of wearable devices in tracking sleep patterns rather than their successful implementation in new behavioral intervention strategies [6]. Second, most clinical studies assessing sleep quantity and quality to date have relied on either self-reported measures of sleep duration or on polysomnography measurements. Poly- somnography remains the gold standard for sleep measurement, but its use is currently only feasible in a laboratory setting, and studies typically use only 1 or 2 nights of measurement. Self-reported sleep measurements limit the generalizability of any results because of errors due to reporter bias.

This study has limitations. First, it was conducted within a single health system in a clinical trial, and we limited the sample to those patients who obtained sleep baselines (approximately 94% of patients). Second, the trial focused more on physical activity than on sleep. Third, we were not adequately powered to perform evaluations of differences in sleep patterns. Fourth, we did not have qualitative data on patient perspectives of barriers and facilitators to using these devices to monitor sleep. Fifth, while the wearable device used has been found to be accurate for monitoring sleep duration [11], we did not validate it in this study. Sixth, further work is needed to understand what proportion of days devices need to be used to provide robust assessments of sleep patterns.

### Conclusion

In conclusion, a high proportion of patients with ischemic heart disease used wearable devices to track sleep patterns over a 24-week period. Use declined over time but varied based on patient characteristics and was greater in the intervention group than in control. Future research should consider larger evaluations combining wearable devices with behavioral interventions to test ways to risk stratify patients and improve sleep patterns.

## Abbreviations

ACTIVE REWARD: A Clinical Trial Investigating Effects of a Randomized Evaluation of Wearable Activity Trackers with Financial Rewards
